# Investigation of the Optimal Operating Position of an Air Cleaner in Terms of Indoor Air Quality in a Four-Bed Hospital Ward

**DOI:** 10.3390/toxics10070360

**Published:** 2022-06-30

**Authors:** Jungsuk Lee, Su-Hoon Park, Ik-Hyun An, Young-Won Kim, Se-Jin Yook

**Affiliations:** 1 School of Mechanical Engineering, Hanyang University, Seoul 04763, Korea; ljsghs@nate.com (J.L.); parksunekk28@naver.com (S.-H.P.); aih2616@naver.com (I.-H.A.); 2 Green Energy & Nano Technology R&D Group, Korea Institute of Industrial Technology, Gwangju 61012, Korea; ywkim@kitech.re.kr

**Keywords:** indoor air quality, air cleaner, ventilation system, hospital ward, age of air

## Abstract

The use of air cleaners indoors has increased with the increase in indoor activities driven by the COVID-19 outbreak. In this study, the indoor air quality was determined at the location of each patient’s respirator in a four-bed hospital ward equipped with a ventilation system and curtains, by varying the position of one air cleaner. By operating the air cleaner alone without the ventilation system, it was confirmed that it is better to place the air cleaner close to the center of the ward, regardless of whether curtains are used. It was further identified that the farther away the air cleaner is from the center, the worse the age of air could be, compared to the case of operating it in the center. Moreover, the situation where the ventilation system and air cleaner were operated simultaneously in the hospital ward was considered. It was discovered that operating the air cleaner close to the ventilation inlets in the absence of curtains helps to improve the indoor air quality. Furthermore, it was found that the age of the air is generally low near the location where the air cleaner is operated in the presence of curtains. Selecting an optimal position for the air cleaner can improve the air quality at the location of each bed in a four-bed hospital ward.

## 1. Introduction

In recent years, people have spent more time indoors due to the COVID-19 outbreak and the increasing concentration of fine dust in the atmosphere. Consequently, the increase in peoples’ indoor activities can increase the concentration of fine dust indoors [1]. Fine dust can deposit in human respiratory organs and adversely affect human health [2,3]. Various ventilation methods can lower the concentration of fine dust indoors. Natural ventilation can be achieved by simply leaving the door and window open. However, when the concentration of fine dust in the atmosphere is high, natural ventilation can increase the concentration of fine dust in the room [4,5]. Several studies have been conducted to improve indoor air quality by applying a mechanical ventilation method that introduces external air into the room or discharges indoor air to the outside using a ventilation system installed in a fixed location. The concentration of particles in the building through mechanical and natural ventilation has been measured, and it has been confirmed that mechanical ventilation can reduce the effect of atmospheric particle concentration compared with natural ventilation [6]. The size and number concentration of particles in an office were measured based on the flow pattern of the ventilation system, and it was confirmed that the change in the number concentration of particles based on particle size varies according to the flow pattern [7]. The ventilation system was operated in an ambulance to observe the number concentration of particles generated by a patient’s cough, and it was confirmed that the higher the flow rate of the ventilation system, the faster the decrease in the particle number concentration [8]. The particle removal efficiency by two ventilation methods, i.e., mixing and displacement ventilation, was examined, and the mixing ventilation method was found to be more effective in particle removal [9].

The ventilation system filters the air introduced from the outside using a filter, and thus, supplies cleaner air indoors compared to natural ventilation. However, because the ventilation opening has a fixed position, the air quality of multi-use facilities such as hospitals varies based on the location. However, an air cleaner can be moved and installed at an arbitrary location to lower the concentration of fine dust in a room. For this reason, the use of air cleaners to improve indoor air quality is increasing, and many studies have been conducted on the effect of using air cleaners on indoor air quality. The effect of the discharge direction of the air cleaner was investigated, and it was confirmed that the particle number concentration decreased when the air cleaner discharged upward and horizontally [10]. Several air cleaners were operated in a classroom to observe the indoor particle number concentration, and it was confirmed that the particle number concentration decreased rapidly when three air cleaners were used simultaneously [11]. The particle removal performance of the air cleaner was evaluated according to the size of the indoor space, and it was confirmed that, even when the same air cleaner was used, the particle removal performance decreased as the size of the indoor space increased [12]. The particle number concentration was monitored when the air cleaner was used in an office where several pieces of furniture were arranged, and it was confirmed that the indoor particle removal performance decreased when the filter efficiency of the air cleaner deteriorated [13]. The PM_2.5_ concentration when cooking was observed based on whether the air cleaner was used indoors, and it was confirmed that the concentration of PM_2.5_ in the room could be significantly lowered when using the air cleaner [14]. The indoor fine dust concentration can be greatly reduced using an air cleaner, as proved in the previous literature [11,12,13,14]; however, there are only a few studies on how the fine dust concentration varies depending on the operating position of the air cleaner in the indoor space.

The probability of infection by the SARS-CoV-2 virus can increase depending on the concentration of fine dust [15,16,17]. The fine dust in a hospital can be a source of viruses for occupants including patients [18,19], and thus, there is an urgent need for studies on reducing the fine dust concentration in hospitals where patients spend most of their time. In this study, using an additional air cleaner was considered to improve indoor air quality in a four-bed hospital ward equipped with a ventilation system. The age of air is an indicator used to compare indoor air quality [20,21,22], and it has been found that there is a close relationship between changes in particle number concentration and changes in the age of air [23]. Previous studies have shown that the overall indoor air quality in the ward can be greatly improved by operating an air cleaner along with the ventilation system and that the age of air can deteriorate depending on the position in the ward due to the flow interference [24,25]. Therefore, in this study, the change in the age of air based on the location of the air cleaner in a four-bed hospital ward was investigated. It was further investigated how the age of air at the patient’s respirator position varies depending on the location of the air cleaner in a situation where the patient lies in bed for a long time.

## 2. Experimental Method

**Figure 1:**
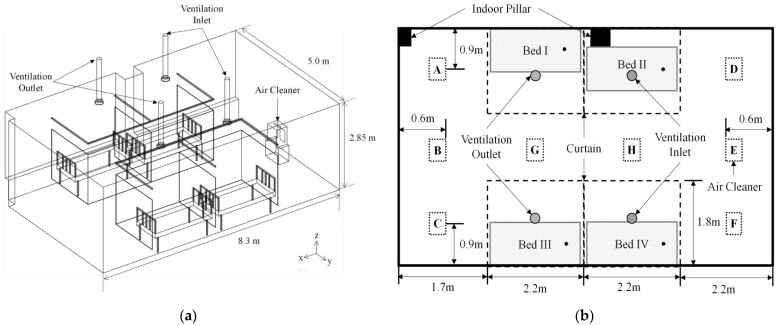
Laboratory configuration. (**a**) Isometric view; (**b**) Plan view.

A laboratory simulating a four-bed hospital ward was constructed, as shown in Figure 1. The size of the laboratory was 8.3 m in length, 5.0 m in width, and 2.85 m in height. The laboratory volume was 118.3 m^3^. Four beds were placed in the laboratory. Curtains with a height of 1.65 m were installed at 0.2 m away from each bed for the patients’ privacy. Two ventilation inlets were provided on the ceiling of the Bed II and Bed IV areas and two ventilation outlets on that of the Bed I and Bed III areas. One air cleaner was operated at one of the eight positions (A to H), as shown in Figure 1b. The positions of A, C, D and F were arranged such that they were on the same line in the *x*-direction as the center of the nearby ventilation inlet or outlet. The positions B, E, G and H were arranged in the center of the interior in the *y*-direction; in particular, the positions of G and H were arranged on the same line as the ventilation outlets and inlets in the *y*-direction, respectively. The air cleaner sucked air horizontally and discharged it vertically towards the ceiling. The flow rate of the ventilation system or the air cleaner was measured using a hood-type airflow meter (Testo 420, Testo, Inc., Lenzkirch, Germany). The total flow rates of the ventilation system and the air cleaner were measured to be 434 m^3^/h and 288 m^3^/h, respectively. The ventilation system and the air cleaner were equipped with a H14 class HEPA filter and an E11 class HEPA filter, respectively. Based on the results of measuring the collection efficiency of the filter used in each airflow generator, the collection efficiency was determined to be 99.99% for the filter of the ventilation system and 95% or higher for that of the air cleaner.

**Figure 2:**
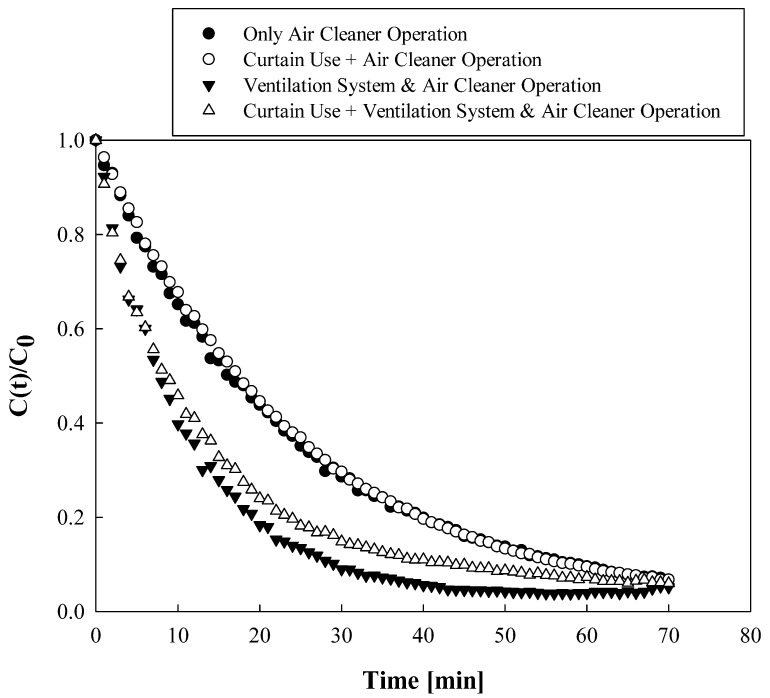
Experimental results of the particle number concentration over time.

The particle number concentration was measured at the patient’s respirator location, considering the situation in which the patient lies on each bed. The inlet of the sampling probe, denoted by a black dot on each bed in Figure 1b, was placed 0.85 m above the floor. The flow rate of the aerosol sampled at each position was 1 L/min. The particle number concentrations at the beds were monitored using Condensation Particle Counters (CPC; Model CPC-0701, HCT Co., Ltd., Icheon, Korea). After opening the window for 30 min to ventilate the ward sufficiently, the windows were closed and incense was burned to generate particles. When the indoor particle number concentration was over 10^5^ cm^−3^, the incense was extinguished and a fan was used to spread the particles evenly in the ward. When the indoor particle number concentration distribution became uniform, i.e., when the particle number concentrations at the monitoring locations were almost equal, the fan was turned off, and the air cleaner and/or ventilation system were/was operated. After more than 10 min for the indoor airflow to stabilize, the change in particle number concentration over time was measured using CPC. The temperature range was 18–22 °C and the relative humidity (RH) range was approximately 40–60%. Figure 2 shows a few examples of the experimental results of the particle number concentration over time. The vertical axis value was normalized by the particle number concentration value corresponding to the initial time (0 min) for each experimental condition.

When the air cleaner and/or ventilation system were/was operated, the indoor air was filtered by the filter used in each device, and thus, the particle number concentration decreased over time, and the decrease rate varied depending on the experimental conditions; however, it reduced exponentially in all cases. Therefore, the curve fitting method was applied to the experimental data using Equation (1) [23,25].

(1) $$C\left(t\right)=y_{0}+A\exp\left(-\frac{t}{\tau }\right),$$

where *t* is the time, *C*(*t*) is the particle number concentration at time *t*, *y*_0_ is the particle number concentration converging after a long time, *A* is the initial particle number concentration, and *τ* is the result obtained through the curve fitting, which represents the age of air. The age of air has a small value when the particle number concentration decreases rapidly at the corresponding position and a high value when the particle number concentration decreases slowly. The experimental result of the age of air was found to be repeatable with an error of less than 5%, in the temperature range of 18–22 °C and the RH range of 40–60%.

## 3. Numerical Method

As shown in Figure 1a, we set a calculation area for simulation and performed flow analysis using ANSYS FLUENT Release 16.1, a commercial computational fluid dynamics (CFD) code. The flow was assumed to be three-dimensional, steady, incompressible, and turbulent. The *k-ε* model for turbulent flow analysis was used [23,24,25]. The boundary conditions for the flow analysis were as follows: The velocity inlet conditions were set in the ventilation inlets for introducing air into the ward and the outlets of the air cleaner; The flow rates were set to 3.92 m/s and 4.78 m/s, respectively; The pressure outlet conditions were applied to ventilation outlets and air cleaner suction parts that inhale air from the indoors, and mass flow rates for each were set to 0.074 kg/s and 0.098 kg/s, respectively. The temperature field was not analyzed because there was no significant difference between the temperature of the air flowing into the ward through the ventilation system and the air cleaner and the indoor temperature.

When constructing the grids, considering the complex shapes of the ventilation inlets and outlets, a tetrahedral mesh was used in the area surrounding these and a hexahedral mesh in all other areas. As a result of conducting the grid independence test, the number of grids was determined as between 12 and 14 million. When the relative error of all equations was less than 10^−5^, the calculation was completed. The age of air was calculated using the following equations [23,25]:

(2) $$\frac{\partial }{\partial x_{i}}\rho u_{i}\Phi -\dot{J}\frac{\partial \Phi }{\partial x_{i}}=\rho ,$$

(3) $$\dot{J}=-\left(\rho D_{m}+\rho D_{t}\right)\frac{\partial \Phi }{\partial x_{i}},$$

where *ρ* is the air density, *u_i_* is the flow velocity, *j* is the diffusion rate of the air, *D_m_* is the molecular diffusivity, *D_t_* is the turbulent diffusivity, and *Φ* is the age of air. The collection efficiencies of filters used in the ventilation system and the air cleaner were 99.99% and >95%, respectively; thus, the age of air was set to 0 in the ventilation inlets and the air cleaner discharge part, and the flux value of the age of air in the ventilation outlets and the air cleaner suction unit was set to 0.

Table 1 lists 26 cases based on the installation position of the air cleaner, operation of the ventilation system, and use of curtains. In Cases 1–10, only the air cleaner was operated and the ventilation system was turned off. In these cases, the positions of D, E, and F were not considered due to the symmetry of the flow pattern based on the installation position of the air cleaner. In Cases 11–26, the air cleaner and the ventilation system were operated simultaneously; since the flow discharged from the air cleaner was affected by the flow caused by ventilation inlets and outlets, all positions from A to H were considered. Meanwhile, bed curtains were not used for Cases 1–5 and 11–18, whereas they were considered for Cases 6–10 and 19–26.

## 4. Results and Discussion

**Figure 3:**
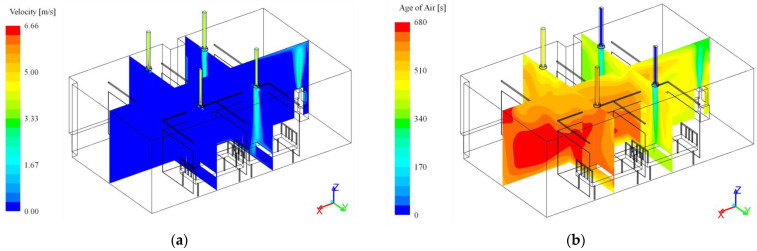
Numerical analysis results of Case 23. (**a**) Velocity distribution; (**b**) Age of air distribution.

Figure 3 illustrates an example of the numerical analysis result. Figure 3a,b presents the velocity and age of air distribution for Case 23, respectively. Figure 3 shows the flow of clean air supplied to the ward through the ventilation inlets and the discharge port of the air cleaner and the flow of the air discharged through the ventilation outlets. Curtains were used for each bed in Case 23; thus, as shown in Figure 3b, the age of air was found to be low in the regions of Bed II and Bed IV located below the ventilation inlets and high in the regions of Bed I and Bed III located below the ventilation outlets. Meanwhile, outside the curtain, the age of air was relatively high in the area near the ventilation outlets, including the positions A, B, C and G.

**Figure 4:**
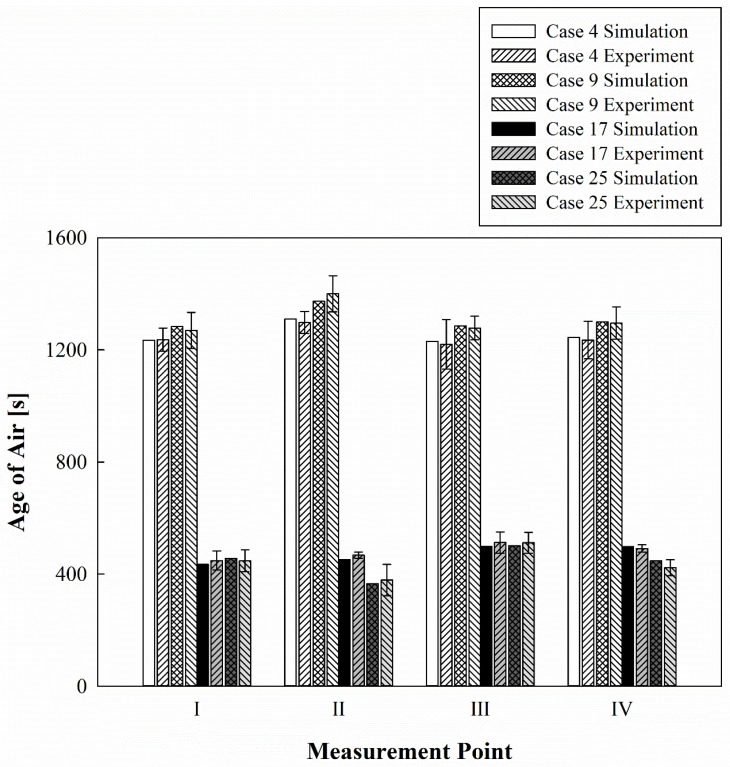
Comparative verification results of experimental measurements and numerical analysis.

**Figure 5:**
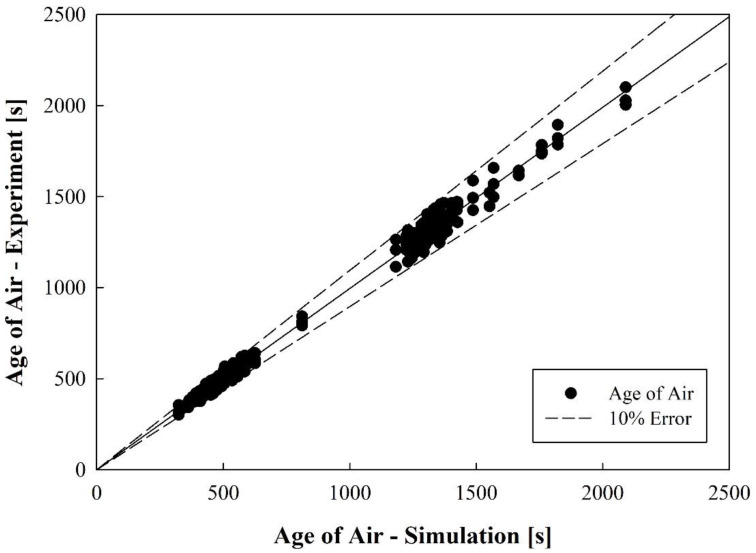
Comparison of the age of air between experimental measurements and numerical analysis.

Figure 4 shows the comparative results of the experiment and simulation for the age of air at the respirator position for patients lying in each bed. Cases 4, 9, 17, and 25 all correspond to situations in which the air cleaner operated at G. In Case 4, the air cleaner was operated without opening the curtain. In Case 9, the air cleaner was operated while opening the curtain. In Case 17, the ventilation system and the air cleaner were operated simultaneously without opening the curtain. In Case 25, the ventilation system and the air cleaner were operated simultaneously while opening the curtain. For all bed positions, when only the air cleaner was operated, the age of air was slightly lower when the curtains of each bed were folded than when they were opened. The age of air was significantly lower in all beds when the air cleaner and the ventilation system were operated simultaneously than when only the air cleaner was operated. In Bed I and III below the ventilation outlets, the age of air difference according to the use of curtains is minimal; however, in Bed II and IV below the ventilation inlets, the age of air was slightly lower when the curtains were opened than when they were folded. The experiment was performed on all 26 cases considered, including the comparison result shown in Figure 4. The error between the simulation and the experiment appeared to be within ±10%, as displayed in Figure 5, when all data for 26 cases were compared. Consequently, it was confirmed that the prediction accuracy of the simulation method used in this study was high, and thus, the analysis was conducted using only the simulation results in the following sections.

**Figure 6:**
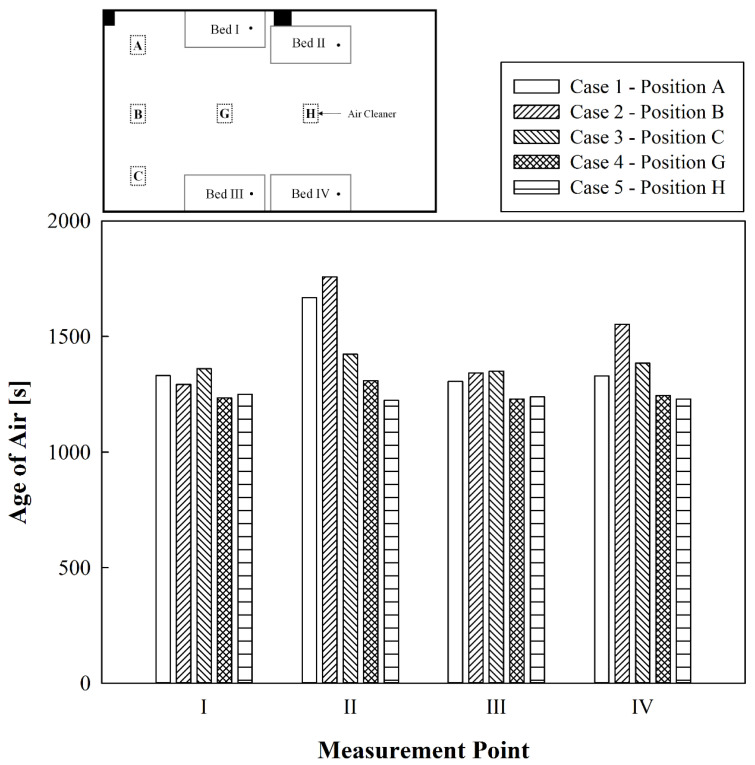
Comparative results of the age of air at locations where only the air cleaner was operated.

Figure 6 shows the comparative results of the age of air at the respirator positions of patients lying in each bed when only one air cleaner was operated at A, B, C, G, or H while the ventilation system and the curtains were not used. When the air cleaner was operated at C, the age of air was similar at all beds; it was slightly higher at Beds II and IV, which were relatively far from the air cleaner than Beds I and III. This is because the air discharged from the air cleaner rose upward and spread throughout the ward, causing strong eddy currents near the two walls perpendicular to each other at the edge of the ceiling. When the air cleaner was operated at A, which was symmetrical to C, the age of air was similar at Beds I, III, and IV; however, it was particularly high at Bed II. This is because the air discharged from the air cleaner was blocked by a pillar immediately next to Bed II and did not reach the respirator position of the patient in Bed II well. This suggests that the presence of obstacles such as pillars or closets that interfere with the flow around the bed may deteriorate the air quality around it. When the air cleaner was operated at B, at the center of the short indoor wall, the age of air was similar at Beds I and III close to the air cleaner; however, they appeared to be relatively high at Beds II and IV that are far from the air cleaner compared with the operating locations of A and C. This is because the air discharged from the air cleaner rose and moved along the ceiling towards the center of the ward, forming a large recirculation flow such that it could not smoothly reach Beds II and IV. When the air cleaner was operated at G or H, the age of air was lower than when it was operated at other positions and similar at all beds. This is because the air discharged from the air cleaner hit the center of the ceiling and spread evenly in all directions. When comparing the results of the operating positions G and H, the age of air was slightly higher only in Bed II when the air cleaner was operated at G, and the others were approximately equal due to the pillar next to Bed II. Therefore, if only one air cleaner is operated with the curtains folded in a four-bed hospital ward without a ventilation system, it is most effective to keep the air cleaner in the center of the ward, while the next best location is to put it at the corner, but not at a position near the center of the wall.

**Figure 7:**
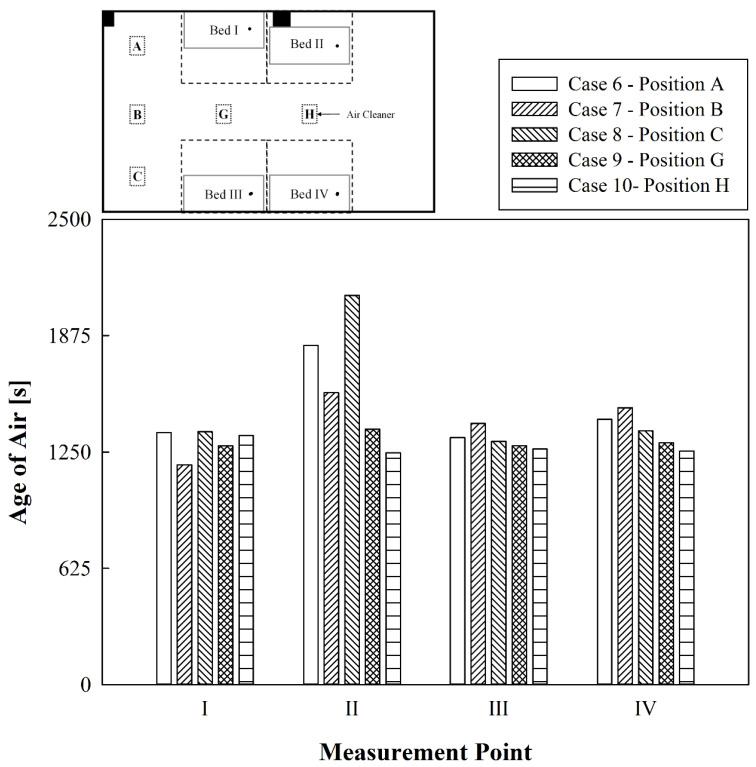
Comparative results of the age of air at locations where only the air cleaner was operated while using curtains.

Figure 7 compares the age of air at the respirator positions of patients lying in each bed when the ventilation system is not running and only one air cleaner is operating at A, B, C, G, or H with all curtains open. When the air cleaner was operated at A or C, the age of air was similar at Beds I, III, and IV; however, it was noticeably high at Bed II. Furthermore, the average values of the age of air were 1569 s and 1512 s, which were the highest among Cases 6–10; this is because the clean air discharged upwards from the air cleaner, moved along the wall in the space between the curtain and the ceiling, and the flow of air was interrupted by a pillar next to Bed II. When the air cleaner was operated at B, the age of air was low in the order of Beds I, III, IV, and II. The age of air at Bed II was the highest because the pillar next to Bed II prevented the clean air from being transferred to Bed II. However, the age of air at Bed I was the lowest because the flow of air blocked by the pillar was recirculated within the Bed I area. Meanwhile, when the air cleaner was operated at G or H, the age of air at all beds was similar because the clean air discharged from the air cleaner spread along the ceiling in all directions, and the average values of the age of air were 1291 s and 1259 s, which were the lowest among Cases 6–10. However, due to the influence of the pillar next to Bed II, when the air cleaner was operated at G, the age of air at Bed II was slightly higher than in other places, and when the air cleaner was operated at H, the age of air at Bed I was slightly higher than in other positions. Therefore, when using curtains in a four-bed hospital ward without a ventilation system, the air cleaner should preferably not be operated at the indoor corner positions; it is better to operate it close to the indoor center.

**Figure 8:**
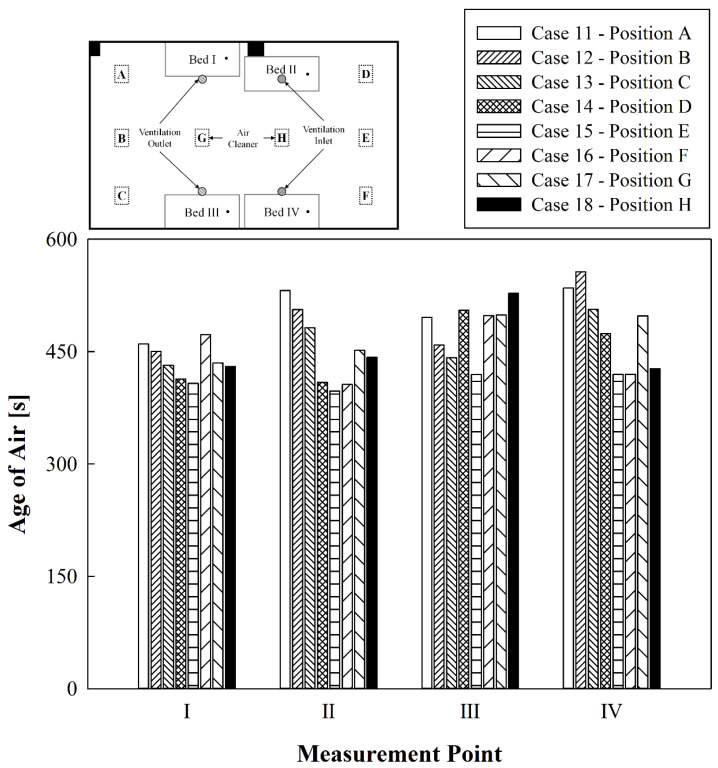
Comparative results of the age of air at locations of the air cleaner while operating the ventilation system.

Figure 8 compares the age of air at the respirator positions of patients lying in each bed when one air cleaner was operated at either A to H with the ventilation system on and the curtains folded. Compared to the results of when only the air cleaner was operated (Figure 6), the age of air greatly decreased in this case due to the simultaneous operation of the air cleaner and the ventilation system. When the air cleaner was operated at A, B, or C by the wall near the ventilation outlets, the values of the age of air at Beds I and III below the ventilation outlets were lower than those at Beds II and IV below the ventilation inlets. This is because the air supplied by the ventilation inlets primarily reaches the respirator positions of patients in Beds II and IV, whereas the air supplied by the ventilation inlets moving towards the ventilation outlets alongside the air discharged from the air cleaners affects the respirator positions of the patients in Beds I and III. Moreover, when the air cleaner was operated at A, B, or C, the age of air at all beds was generally high because the air currents caused by the air cleaner and the ventilation system interfered with each other, preventing clean air from spreading evenly indoors. The age of air was lower in Beds II and IV (below the ventilation inlets) when the air cleaner was operated at D or F (corner positions close to the ventilation inlets) than when the air cleaner was operated at A, B, or C. The age of air was similarly high in Beds I and III (below the ventilation outlets) because the air discharged from the air cleaner does not affect the Beds I and III areas due to interference with the flow of air from the ventilation inlets. The age of air was the lowest at all beds when the air cleaner was operated at E than when it was operated at other positions. This is because the flow of air discharged downwards from ventilation inlets and that discharged upwards from the air cleaner did not interfere; they caused synergies and thereby diffused clean air throughout the ward. Contrarily, when only the air cleaner was operated, G and H were the most suitable installation locations for the air cleaner; however, when the ventilation system and the air cleaner were operated simultaneously, G and H were not the optimal installation locations for the air cleaner. This is because the airflow from the air cleaner and that from the ventilation system interfered with each other; thus, clean air could not be easily diffused into the ward. Therefore, when the ventilation system and air cleaner were operated simultaneously with all curtains folded, the overall age of air in the ward lowered, and it was found that the best way to improve indoor air quality is to operate the air cleaner near the center of the wall close to the ventilation inlets, i.e., at position E.

**Figure 9:**
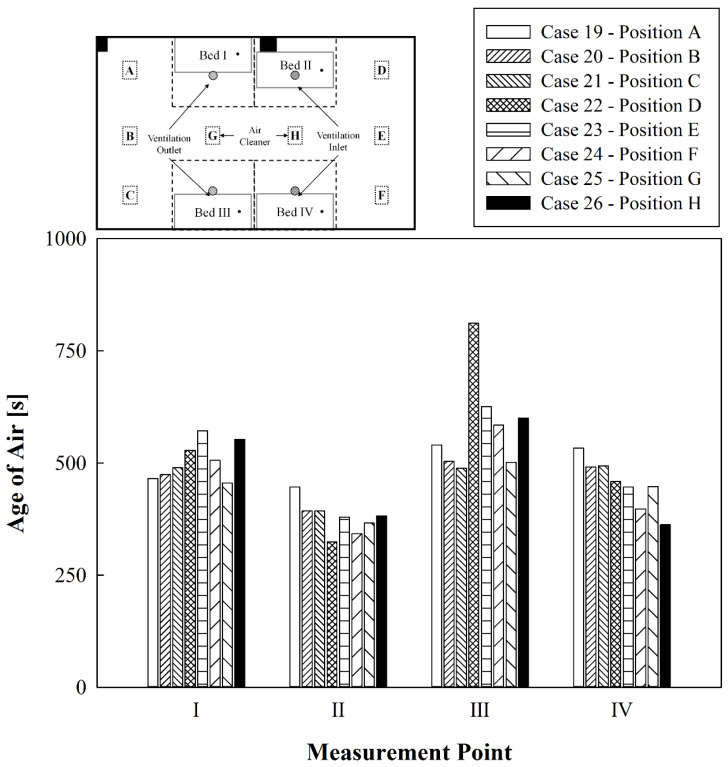
Comparative results of the age of air at locations of the air cleaner with curtain use and ventilation system operation.

Figure 9 shows a comparison of the age of air in the respirator positions of patients lying in each bed when a single air cleaner was operated at either A to H with the ventilation system running and all curtains open. Compared to the results in Figure 7, in which only the air cleaner was operated, the age of air significantly lowered when the air cleaner and the ventilation system were operated simultaneously; however, the results were considerably different from the local age of air distribution tendency of Figure 7. For Beds I and III near the ventilation outlets, when the air cleaner was operated at A, B, C, or G, the air discharged from the air cleaner directly reached the area of Beds I and III due to interference with the airflow from the ventilation inlets, and the age of air was relatively low at Beds I and III. Contrarily, when the air cleaner was operated at D, E, F, or H, the age of air at Beds I and III relatively increased because the airflow path from the air cleaner to Beds I and III was lengthened and the flow was hindered by curtains. Therefore, it is helpful to operate the air cleaner close to the ventilation outlets for lowering the age of air in the bed near the ventilation outlets when the curtains are opened. Meanwhile, irrespective of the operation position of the air cleaner, the age of air was the lowest at Bed II (located below one of the ventilation inlets) due to the recirculation of clean air supplied from the ventilation inlets by the pillars next to Bed II. The age of air in Bed IV under another ventilation inlet was relatively low when the air cleaner was operated at F or H, whereas it was relatively high when the air cleaner was operated at other positions. This is because when the air cleaner was closer to Bed IV, the air discharged from the air cleaner could reach Bed IV via a shorter route. Based on the results shown in Figure 9, it was found that operating the air cleaner in a situation where the ventilation system is operated with all curtains opened helps improve the air quality of the bed located close to the air cleaner.

## 5. Conclusions

In this study, the age of air distribution of each bed was analyzed according to the operation of the ventilation system, the use of curtains, and the operation position of one air cleaner in a four-bed hospital ward. The age of air was determined by experimentally grasping the reduction characteristics of the particle number concentration in the hospital ward. Alternatively, the age of air was calculated through numerical analysis, and the results were compared with the experimental results.

In order to efficiently enhance the overall indoor air quality in the ward, it is important to make the clean air discharged from the air cleaner spread as widely as possible. If the air cleaner discharges air upwards, then the clean air can flow in every direction along the ceiling. Therefore, it was confirmed that, in the case with no curtains or ventilation systems, clean air could be supplied smoothly throughout the ward by operating the air cleaner in the center of the ward compared to near the wall. In the case with all curtains opened and no ventilation system used, it is also better to operate the air cleaner in the center of the ward as in the previous case; however, the use of the curtains slightly increased the age of air.

When the air cleaner is operated along with the ventilation system, the average age of air in the ward can be lower than when the air cleaner or the ventilation system are solely operated. However, it should be noted that the air flow induced by the air cleaner can interfere with the air flow caused by the ventilation system and that the flow interference pattern depends on whether or not the curtains are used. The ventilation system is generally installed on the ceiling. Then, the air is discharged downwards from the ventilation inlets and flows toward the ventilation outlets. If the air cleaner, which discharges air upwards, is placed near a region between the ventilation inlets, then the clean air flow from the ventilation inlets can go through the lower area of the ward, while the clean air flow from the air cleaner can pass though the upper area of the ward, resulting in a synergistic effect. Thus, when the ventilation system was operated with all curtains folded, the indoor air quality improved when the air cleaner was operated near the center of the wall close to the ventilation inlets rather than in the center of the ward. On the other hand, if the curtains are opened, then the clean air flow discharged from the ventilation inlets is disturbed by the curtains and cannot spread widely. In this case, the air cleaner is needed to be placed where the clean air from the ventilation inlets cannot reach effectively. Thus, when the ventilation system was operated with all curtains opened, the age of air was generally low in a bed close to the location where the air cleaner was operated.

It was confirmed that the flow of clean air discharged from the air cleaner interacted with the airflow by the ventilation system or was affected by obstacles such as curtains or walls; thus, the local distribution of age of air varied greatly. Therefore, further research should be carried out to consider various cases of the air supply/exhaust location of the ventilation system and to find the optimal location for operating the air cleaner according to various arrangements of indoor furniture that may affect airflow. Furthermore, it is necessary to review the use of several air cleaners in a hospital ward with a large number of patients.

## Figures and Tables

**Table 1:** Operation cases based on the air cleaner position.

Case	Cleaner Position	Ventilation	Curtain
1	A	Off	Folded
2	B		
3	C		
4	G		
5	H		
6	A	Off	Opened
7	B		
8	C		
9	G		
10	H		
11	A	On	Folded
12	B		
13	C		
14	D		
15	E		
16	F		
17	G		
18	H		
19	A	On	Opened
20	B		
21	C		
22	D		
23	E		
24	F		
25	G		
26	H		

## Data Availability

Not applicable.
